# Supplementary material for: Therapeutic Outcomes and Prognostic Factors of Unresectable Intrahepatic Cholangiocarcinoma: A Data Mining Analysis
Source: J Clin Med. 2021 Mar 2;10(5):987. doi: 10.3390/jcm10050987 (PMC7957874; doi:10.3390/jcm10050987)
Supplement: Supplementary file 1 [file jcm-10-00987-s001.pdf]

supplementary Table S1. Baseline characteristics of 8 patients who have not been include

Factor	Number or Median (Range)
Age (years)	69(52-75)
ECOG PS 0/1/2	4/3/1
Sex Male/Female	7/1
Etiology HBV/HCV/non-B, non-C	1/2/5
Stage II/III/IVA/IVB	3/1/1/3
Tumor size (mm)	40 (26-100)
Total bilirubin (mg/dL)	0.81 (0.44-3.08)
AST (U/L)	38 (18-119)
ALT (U/L)	35 (14-52)
LDH (U/L)	212 (161-1188)
γ-GTP (U/L)	125 (33-302)
ALP (U/L)	386 (160-1148)
Albumin (g/dL)	3.61 (2.22-4.29)
BUN (mg/dL)	15.7 (8.8-27.8)
Creatinine (mg/dL)	0.72 (0.48-1.22)
CRP (mg/dL)	1.08 (0.04-11.51)
Sodium (mEq/L)	140 (133-143)
Potassium (mEq/L)	4.1 (3.9-5.1)
Chloride (mEq/L)	105 (99-109)
Hb (g/dL)	13.4 (6.3-15.9)
White blood cell (/μL)	5750 (3700-1290)
Neutrophils (/μL)	3394.4 (1776-11184.3)
NLR	2.25 (1.4-10.6)
CEA (ng/mL)	3.1 (2.1-109.3)
CA 19-9 (U/mL)	183.7 (10.4-87208.9)

Abbreviations: ALP; alkaline phosphatase, ALT; alanine aminotransferase, AST; aspartate aminotransferase, BUN; blood urea nitrogen, CRP; C reactive protein, HBV; hepatitis B virus, HCV; hepatitis C virus, LDH; lactate dehydrogenase, Hb; hemoglobin, NLR; neutrophil-to-lymphocyte ratio, WBC; white blood cell, γ-GTP; gamma-glutamyl transpeptidase
